# Winter activity of *Ixodes ricinus* in Sweden

**DOI:** 10.1186/s13071-023-05843-9

**Published:** 2023-07-10

**Authors:** Petter Kjellander, Ulrika A. Bergvall, Jan Chirico, Karin Ullman, Madeleine Christensson, Per-Eric Lindgren

**Affiliations:** 1 Grimsö Wildlife Research Station, Department of Ecology, Swedish University of Agricultural Sciences, Riddarhyttan, Sweden; 2 Department of Microbiology, National Veterinary Institute (SVA), Uppsala, Sweden; 3 Department of Biomedical and Clinical Sciences, Division of Inflammation and Infection, Linköping University, Linköping, Sweden; 4 Laboratory Medicine, Microbiological Laboratory, County Hospital Ryhov, Jönköping, Sweden

**Keywords:** *Ixodes ricinus*, Climate, Winter activity, Roe deer

## Abstract

**Background:**

In Europe, *Ixodes ricinus* (Acari: Ixodidae) is the most widespread and abundant tick species, acting as a vector for several microorganisms of medical and veterinary importance. In Northern and Central Europe, the tick has a bimodal activity pattern consisting of a peak in spring to the beginning of summer and a second peak at the end of summer. However, several findings of ticks on animals during winter have been reported, which raises the question of whether this is an overwintering strategy or whether ticks are active during winter in Scandinavia. The objectives of our study were to determine (i) whether ticks were active and finding hosts during winter, (ii) whether they parasitize their hosts, and (iii) what climatic factors—i.e., temperature, snow depth and precipitation—govern tick winter activity.

**Methods:**

Throughout three winter seasons, we examined wild-living and free-ranging roe deer (*Capreolus capreolus*) for ticks on 332 occasions. In total, 140 individual roe deer were captured in two climatically contrasting sites in south-central Sweden, Grimsö and the Bogesund research area, respectively. We re-examined individual roe deer up to 10 times within the same winter or approximately once a week (mean 10 days, median 7 days between re-examinations) and recorded the absence or presence of ticks on the animals, and tested to what extent meteorological factors affected tick activity. To determine the attachment day, we used the coxal/scutal index of 18 nymphs and 47 female ticks.

**Results:**

In total, 243 *I. ricinus* were collected from 301 roe deer captures between 14 December and 28 February at the Bogesund study site during three subsequent years (2013/2014–2015/2016). We found attached ticks every third to every second examination (32%, 48% and 32% of the examinations, respectively). However, we collected only three *I. ricinus* females from 31 roe deer captures at the Grimsö study site between 17 December 2015 and 26 February 2016. At the Bogesund study site, based on 192 captures of previously examined deer, we collected 121 ticks, and ticks were found at 33%, 48% and 26% of the examinations during the respective winters. The probability of finding an attached tick on a roe deer at a temperature of −5 °C was > 8% ± 5 (SE), and that probability increased to almost 20% ± 7 (SE) if the air temperature increased to 5 °C.

**Conclusions:**

To the best of our knowledge, this is the first time that winter-active nymphs and female ticks have been documented to attach and feed on roe deer during winter (December to February) in Scandinavia. The main weather conditions regulating winter activity for females were temperature and precipitation, and the lowest estimated air temperature for finding an active tick was well below 5 °C. The behaviour of winter-active and blood-feeding ticks was documented over several winters and in two contrasting areas, implying that it is a common phenomenon that should be investigated more thoroughly, since it may have important consequences for the epidemiology of tick-borne pathogens.

**Supplementary Information:**

The online version contains supplementary material available at 10.1186/s13071-023-05843-9.

## Background

In Europe, *Ixodes ricinus* (Acari: Ixodidae) is the most widespread and abundant tick species acting as a vector for several infectious agents of medical and veterinary importance, including tick-borne encephalitis virus (TBEV), *Borrelia* spp., *Anaplasma* spp., *Rickettsia* spp., *Ehrlichia* spp., *Bartonella* spp., *Francisella tularensis*, *Coxiella burnetii* and eukaryotic protozoans of *Babesia* spp. [1–6]. The natural distribution of the tick ranges latitudinally from North Africa to Scandinavia, and longitudinally from Ireland to Russia [7]. *Ixodes ricinus* is also known as the castor bean tick [8], the cattle tick [9] or the sheep tick [10]. It is a three-host tick, where the adults and nymphs mostly parasitize medium-sized and large animals, while larvae are mainly found on small to medium-sized mammals. However, all developmental stages may occasionally be found on any large, medium-sized, or small vertebrate hosts [11]. The host-seeking activity of the tick has been studied because of its importance as a vector for human and animal pathogens [12, 13]. The seasonal and questing activity of *I. ricinus* seems to be influenced by macro- and microclimatic conditions [14, 15], and several studies have investigated the seasonal activity mainly between March and November [16, 17]. In Northern and Central Europe, the bimodal activity pattern consists of one peak in the spring to the beginning of summer, and one activity peak at the end of summer [18]. In the southern parts of Europe, ticks have been found to be questing throughout the winter [8]. In Fennoscandia, there are to our knowledge no published recordings of questing *I. ricinus* between December and February. Nevertheless, several findings of ticks on animals in winter have been reported, raising the question of whether this is an overwintering strategy or whether ticks are active and questing for any other reason during winter in Fennoscandia [19].

The mechanisms regulating the seasonal activity of *I. ricinus* are likely governed by low temperatures, i.e. freezing combined with high precipitation [18]. Ambient conditions that favour questing activity, such as temperature, relative humidity (RH) and precipitation, have been used as explanatory factors for the presence of questing ticks [17, 19]. However, the importance of these factors varies between study sites and habitats [18]. In south-central Sweden, *I. ricinus* have been found to be active and questing from early spring (March–April) to late fall (October–November) [19]. In Central Europe, a similar seasonal pattern has been found, with just a slightly shorter inactivity period during winter, probably due to higher average temperatures; the tick is thus active from March to November [17]. Randolph et al. [20], on the other hand, recorded nymph and female activity in January and February as well during mild winters in the UK. Studies suggest that *I. ricinus* requires temperatures above 5 °C [21] or even above 7 °C [22] to be active, and this corresponds roughly to the meteorological definition of spring onset and ending of autumn. Tomkins and co-authors [23] found that tick populations living in warm climates start questing at higher temperatures than populations from cooler areas. Such populations living in low-temperature areas responded more quickly to changes in temperature than ticks living in warmer climates [23]. Additionally, the interaction between the tick and its microbiome, including the tick-borne pathogens, can affect tick biology in many ways [24, 25] that could be advantageous to the host in a cold climate. For example, *Ixodes scapularis* has demonstrated increased cold resistance and activity when infected with *Anaplasma phagocytophilum* [26], and higher energy reserves were observed in *I. ricinus* infected with *Borrelia burgdorferi* sensu lato [27]. Scandinavian winters have periods with temperatures above 5 °C that may induce questing activity regardless of latitude. If *I. ricinus* is active and host-seeking during Scandinavian winters, the occurrence will most likely be highly patchy and dependent on the local microclimate [28, 29]. Because of snow cover or high RH, studies of tick winter activity in Scandinavia using dragging or flagging surveys are not possible [30]. Thus, sentinel animals have been suggested as an excellent indicator in presence/absence studies [31]. This was applied in our study by repeatedly examining individual wild-living roe deer (*Capreolus capreolus*) throughout three winter seasons. The objectives of the study were to determine (i) whether ticks were active and finding hosts during winter, (ii) whether they parasitize their hosts, and (iii) what climatic factors, i.e., temperature, snow depth and precipitation, govern winter activity. We assume that if a deer that was previously examined without ticks was later found with a parasitizing tick, the tick must have been active between those two dates and prior to when we found it parasitizing.

## Methods

### Study sites

We captured and individually marked roe deer throughout three winters and re-examined the animals for *I. ricinus* on 1–10 occasions every winter. Over three winters we examined free-ranging roe deer for ticks on 332 occasions. In total, 140 different individuals were captured in two areas with contrasting winter conditions. Roe deer were examined at one coastal area close to Stockholm, the Bogesund research area (59°24′N, 18°12′E). The other study site, the Grimsö Wildlife Research Area (59°40′N, 15°25′E), is an inland area 28 km north and 150 km west of the Bogesund research area.

The Bogesund research area covers 13 km^2^ and consists of approximately 65% forest, mainly dominated by the coniferous species Scots pine (*Pinus sylvestris*) and Norway spruce (*Picea abies*). Common oak (*Quercus robur*), willow (*Salix* spp.) and birch (*Betula* spp.) are also widespread species in deciduous and mixed forest in the area. Agricultural land represents 25% of the area and consists of oats, wheat, rape and hay, where a minor part functions as pasture for horses and cattle. Furthermore, 10% of the area consists of bogs and rocky areas. Herb species are highly represented in most of the habitat types [32, 33]. The climate is characterized by moderate winters and warm, relatively dry summers. The warmest month is July, with an average temperature of 17 °C, and the coldest is February with −3 °C. Annual precipitation is around 520 mm, with most falling in July and least in February. Snow normally occurs during the period of December–March with approximately 50 days of > 10 cm of snow. Based on pellet count surveys [34], the roe deer density in this area was 12.1 roe deer per km^2^ during the period 2013–2015 (Kjellander et al. unpublished data).

The Grimsö Wildlife Research Area covers 130 km^2^ and consists mainly of intensively managed Scandinavian boreal forest dominated by Norwegian spruce (*Picea abies*) and Scots pine (*Pinus sylvestris*)*.* Temperatures normally range from −20 °C in winter up to 25 °C in summer, with an average annual precipitation average of 670 mm. Snow normally occurs during the period from December to early April, with approximately 100 days of > 15 cm of snow. Based on pellet count surveys, the roe deer density in this area was 2.5 roe deer per km^2^ in the year 2013 (Kjellander et al. unpublished data).

### Meteorological data

The meteorological data consisted of the temperature, snow depth (cm) and precipitation (mm), characterized as light rain, rain, sleet and snow. Temperature data and snow depth were provided by the closest weather stations (Swedish Meteorological and Hydrological Institute, SMHI; Creative Commons 4.0) situated 10 km from the Bogesund research area (59°34′N, 18°05′E) and 37 km from the Grimsö Wildlife Research Area (59°93′N, 14°89′E). The accuracy of estimated temperatures for a given location will decrease as the distance from the station increases due to the influence of local terrain, topography and other microclimatic factors; our reported meteorological estimates should be considered as indices.

#### Roe deer capture

Roe deer were captured in a box trap (approved by the Swedish Environmental Protection Agency, registered as “L6 Rådjursfälla M/Öster Malma”). Traps were prepared during sunset, between 15:00 and 17:00, by placing 1 L forage in the trap. The forage consisted of Renfor, 11.4 MJ and 113 g crude protein per kg dry substance (88%), made of corn, milling by-products, sugar beet by-products, minerals, vitamins, fat and vegetable oils, manufactured in Sweden by Lantmännen for reindeer (*Rangifer tarandus*). Roe deer were handled between 07:00 and 09:00 the next morning and immediately released after handling [35]. Animals were recaptured and re-examined approximately once a week (mean 10 and median 7 days between captures).

Ethical permission for handling roe deer was approved by the Ethical Committee of Animal Experiments in Uppsala, Sweden (permits C302/2012 and C149/2015).

#### Ticks

To determine whether the animals had been subjected to “questing” ticks, we recaptured and re-examined the roe deer. By questing we do not necessarily consider ticks to climb onto vegetation for host-seeking; rather, during winter they may be activated quiescent ticks that opportunistically find hosts when conditions are favourable. Attached ticks were detected visually or by hand, i.e. through tactile examination of the ears, neck, groins and belly, and were collected and transferred into vials and stored in −80 °C freezers for later ocular determination to species and development stage under a stereo microscope. The examination of roe deer and collection of ticks were performed over 59 days between 19 December 2013 and 13 February 2014, 15 December 2014 and 27 February 2015, and 14 December 2015 and 26 February 2016, respectively, at the Bogesund research area, and during 17 December 2015 and 26 February 2016, at the Grimsö Wildlife Research Area.

To test to what extent the meteorological factors described above affected the probability of finding a tick attached to a deer, we recorded the absence or presence of ticks at each examination. For each tick, the approximate day it was attached to the roe deer was estimated by counting back from the day the roe deer were caught to the time a tick possibly had attached to the roe deer using the coxal/scutal index (C/S index) [35]. The C/S index is based on the state of engorgement estimated by calculating either the scutal index (the ratio of the width of the scutum to the length of the idiosoma) or the coxal index (the ratio of the width of the scutum to the distance between the basal coxae of the fourth pair of legs—the coxal gap). The C/S index has been suggested to be used to estimate the period of time that ticks removed from patients have been feeding, thus assisting with the assessment of pathogen transmission risk [36]. We estimated the day for attachment for a subset of the ticks collected in the two first winters (2013/2014 and 2014/2015), in total 18 nymphs and 47 females. The subset is based on 103 examinations of roe deer, 40 with ≥ 1 ticks and 63 with no ticks found. If a nymphal or female tick was found to be attached to a roe deer on a given day (*t*_0_) and it was estimated according to the C/S index that the tick had been parasitizing the animal for 48 h and the attachment date was defined to be 2 days back in time (*t*_−2_), and so on. For each found tick, the possible days for it to be active was set to a maximum of 6 days (*t*_−6_) before the capture, since the reliability of the C/S index declines considerably after that time [36]. For example, a roe deer caught in a given day (*t*_0_) had only one attached tick. This tick was using the C/S index estimated to be attached 2 days earlier (*t*_−2_), as previously described. Consequently, and in accordance with this definition, no other ticks could have been attached during the previous 4 days (*t*_−3_ to *t*_−6_) or the day immediately prior to the capture day (*t*_−1_). In this way, a set of presence/absence data for tick activity was created for each captured roe deer, 6 days back in time from the capture (*t*_0_). Each of the unique 6 days’ corresponding meteorological data could then be used to investigate the most likely condition during the estimated attachment day (EAD) or 1 or 2 days prior to EAD, when ticks potentially could have been active and finding the host. This was done because the exact EAD is not necessarily the day the tick was active and found the host.

### Statistical analysis

The generated data consisted of a table with estimated days with presence/absence of active ticks and the meteorological data for each of 6 days prior to a found attached tick. Thus, we included 103 examinations of roe deer to assess whether the probability of finding active ticks of different stages was an effect of weather conditions. We fitted a logistic regression model for the absence or presence of an attached tick at the EAD, 1 day prior to EAD or 2 days prior to EAD. The relevant explanatory variables, temperature, precipitation and present snow depth were selected or not selected by model selection based on the Akaike information criterion (AIC), corrected for small sample sizes, and choosing the model with the lowest AIC_*c*_ value [37]. Models with a small difference, i.e., with a ΔAIC_*c*_ < 2, have substantial support [37]. Therefore, we applied the principle of parsimony and selected the model with the lowest number of parameters. In addition, we calculated the probability of finding actively host-seeking ticks during the most likely (AIC_*c*_ selected) weather conditions.

To avoid effects of collinearity between explanatory variables, we also tested whether precipitation and snow depth on a certain day correlated with temperature on the same day using the Spearman rank correlation. It was found that snow depth but not precipitation was highly correlated with temperature (snow vs temperature: *r*_s_ = −0.43; *t* = 8.78, *P* < 0.001; *N* = 350, precipitation vs temperature: *r*_s_ = 0.019; *t* = 0.35, *P* = 0.73; *N* = 350), and thus snow depth was dropped from further analyses (Additional file 1: Appendix 3). Statistical analyses were performed in R [38] and Statistica 13 (StatSoft 2016).

## Results

In total, we collected 243 *I. ricinus* from 301 roe deer captures between December 14 and February 27, during three consecutive winters (2013/2014, 2014/2015 and 2015/2016 at the Bogesund research area (Tables 1, 2). However, we collected only three *I. ricinus* females from 31 roe deer captured at the Grimsö Wildlife Research Area between 17 December 2015 and 26 February 2016 (Tables 1, 2).

**Table 1:** Summary of the number of feeding ticks found at wild roe deer in the two areas: Bogesund and Grimsö, between 14th December and 28th February

Study site	Bogesund research area				Grimsö research area
Year (winter)	13/14	14/15	15/16	In total	15/16
No. of individual roe deer	30	42	38	110	30
Total number of examinations	37	83	181	301	31
Re-examined roe deer	6	42	144	192	1
Mean examinations/roe deer (min–max)	1.2 (1–3)	2.0 (1–6)	4.8 (1–10)	2.7 (1–10)	1 (1–2)
No. of examinations with ticks	12	40	57	160	3
No. of new ticks (total)	2	38	81	121	0
Proportion of examinations, new ticks (%)	33.3	47.6	25.7	35.8	0
Proportion of examinations, ticks (%)	32.4	48.2	31.5	36.2	0.097

An “examination” refers to a single capture of a roe deer, and each roe deer may have none, one or more ticks. A single roe deer may have been caught, examined, recaptured and re-examined several times. “Re-examination” refers to when a roe deer has previously been trapped in the same winter, caught and examined once again. “New tick” therefore refers to that the tick can be determined as new since the previous capture and examination

**Table 2:** Number of ticks attached to roe deer during three winters at 301 examinations at Bogesund and 31 examinations at Grimsö

Area	Bogesund research area								Grimsö research area	
Year	13/14		14/15		15/16		Over three winters		15/16	
Total amounts	%	Total	%	Total	%	Total	%	Total	%	Total
Nymphs	11.1	2	22.5	16	14.3	22	16.9	40	0	0
Females	61.1	11	53.5	38	59.7	92	58.2	141	100	3
Males attached	22.2	4	19.7	14	25.3	39	22.8	57	0	0
Males free	5.6	1	42.8	3	0.6	1	2.1	5	0	0
Males total	27.8	5	1.4	17	16.7	40	24.9	62	0	0
Total ticks		18		71		154		243		3
Newly attached										
Nymphs	50.0	1	14.3	7	16.0	13	17.4	21		
Females	50.0	1	40.8	20	60.5	49	57.9	70		
Male and female attached	0.0	0	18.4	9	23.5	19	23.1	28		
Males free	0.0	0	4.1	2	0.0	0	1.7	2		
Males total	0.0	0	22.4	11	23.5	19	24.8	30		
Newly attached		2		38		81		121		

**Fig. 1:**
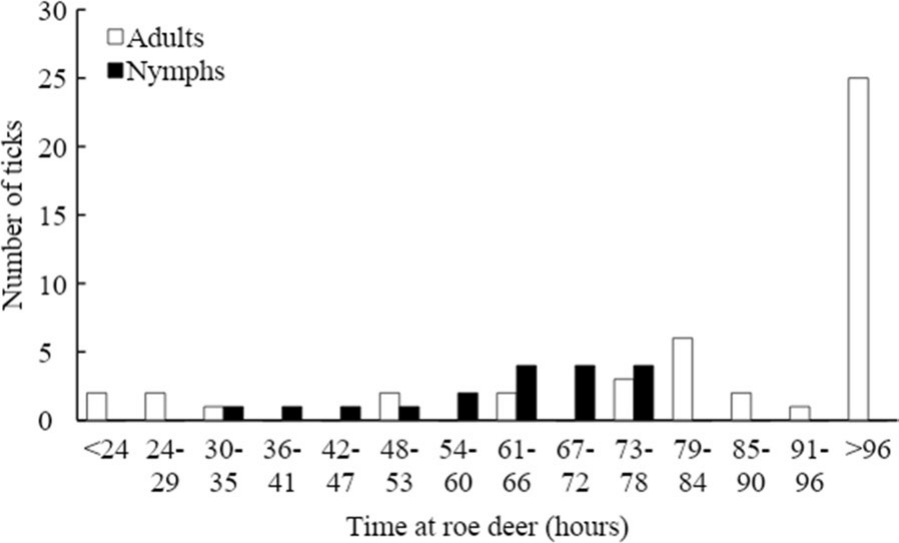
The feeding time in hours for 66 females and 18 nymphs found on roe deer at the Bogesund research area in the winter of 2013/2014 and 2014/2015

We re-examined previously captured and examined roe deer between 1 to 10 times within the same winter season. In 192 captures at Bogesund of previously examined animals, we collected 121 ticks (Table 2). At the Bogesund research area where we examined roe deer for three consecutive winters, we found ticks at 32.4%, 48.2% and 31.5% of the examinations. When we re-examined roe deer within the same winter season, we found newly attached ticks at 33.3%, 47.6% and 25.7% of the examinations, respectively (Table 1, Additional file 1: Appendices 1, 2). We found 18 nymphs and 47 females feeding during 2013/2014 and 2014/2015 (Fig. 1, Table 3). According to the coxal/scutal index calculations, the mean time of feeding until the examination was 2.6 ± 0.5 SD days for nymphs and 3.4 ± 0.9 SD days for females, respectively. After excluding nine females that had been feeding for > 96 h, mean estimated feeding time until examination was 2.9 ± 1.0 SD days (*n* = 56) (Fig. 1, Table 3).

**Table 3:** Estimated mean number of days of attachment and temperature data during 6 days before assumed attachment of 18 nymphs and 38 female ticks on roe deer hosts

	Developmental stage	
	Nymph	Female
Mean time days attached	2.6	3.4
Highest temperature	5.5	7.6
Lowest temperature	−8.5	−6.7
Average temperature	1.9	3.9
Lowest temperature daytime	−3.9	−3.9

The lowest measured air temperature in the vicinity of the Bogesund research area was −13.9 °C at night, and the highest measured daytime temperature was 12.9 °C. Out of the total number of ticks collected, there were 16.9% nymphs, 58.2% females and 24.9% males, whereas the proportion of males found in the fur and not attached was 2.1% (Table 4). None of the ticks from the Grimsö Wildlife Research Area was found on a recently examined roe deer, and thus we cannot be certain that it was attached during the winter. We found ticks during all three winter months at the Bogesund research area, in total 129 ticks (0.33 ticks/roe deer), i.e., in December, 30 ticks (0.56 ticks/roe deer) in January, and in total 67 ticks (1.99 ticks/roe deer) in February (Additional file 1: Appendix 1). Of the three ticks found at the Grimsö Wildlife Research Area, two were found in January and one was found in February (Additional file 1: Appendix 2). The average day temperatures in the vicinity of the Bogesund research area for the different years were 1.2 °C, 3.6 °C and 1.2 °C, respectively. The average day temperature in the vicinity of the Grimsö Wildlife Research Area was 2.4 °C.

**Table 4:** Model selection table for different parameters to explain the estimated day a tick was active and host-seeking in relation to the estimated attachment day (EAD, 0) or 1 (EAD, –1) or 2 days prior (EAD, −2) to the EAD, from 14 December to 28 February

Model	AIC_c_	∆AIC_c_
**EAD, −2 = Temp**	**274.4**	**0**
EAD, 0 = Temp + Temp × Precipitation	275.0	0.6
EAD, −2 = Temp + Temp × Precipitation	276.9	2.5

In bold is the selected and most parsimonious model with the lowest AIC_*c*_ and lowest number of parameters

**Fig. 2:**
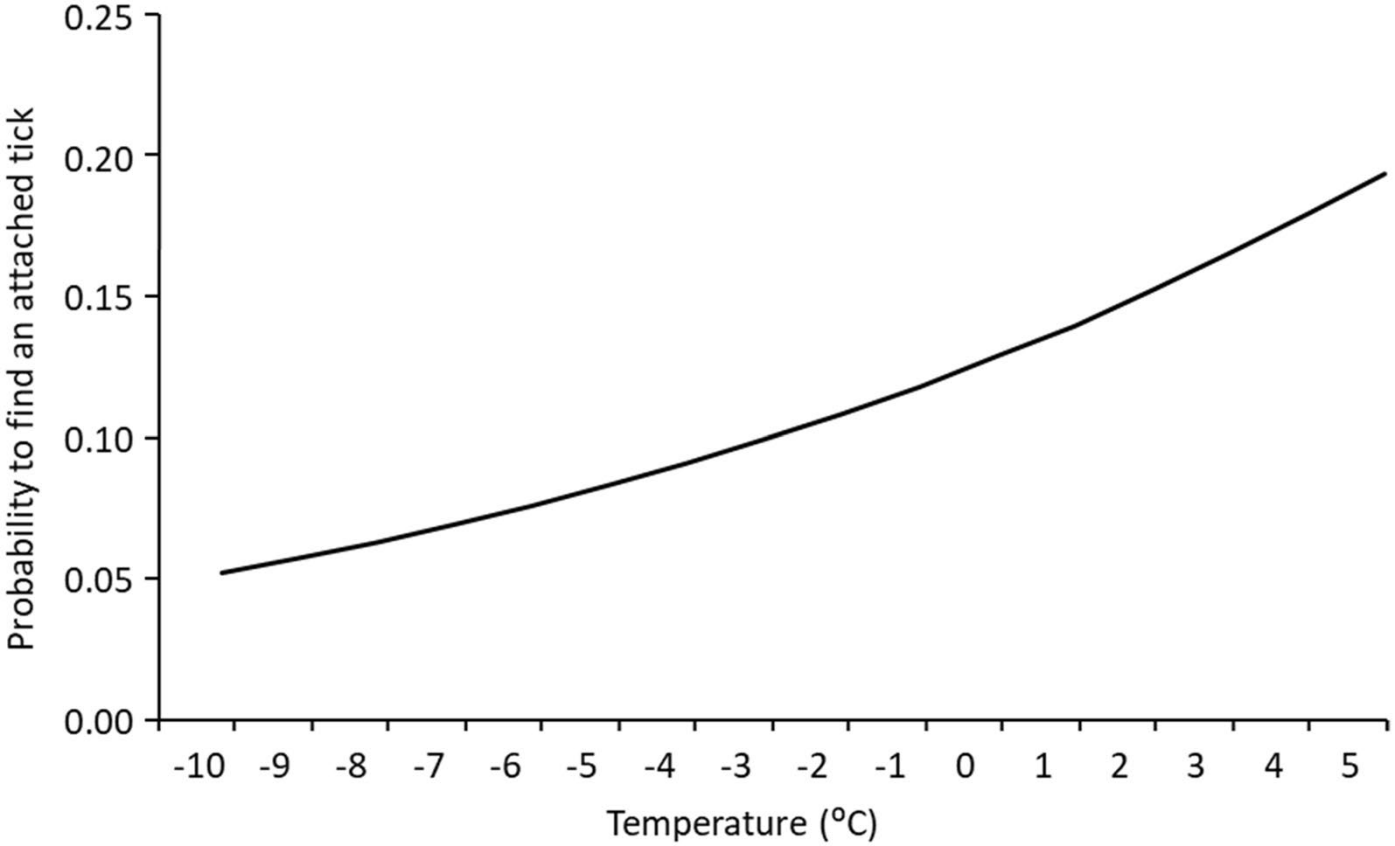
The probability of finding a female tick on a roe deer in relation to air temperature 2 days prior to the estimated attachment day, during the period from December to February 2013–2015 in south-central Sweden

In total, we recorded presence/absence data for 40 examinations where we found one or more ticks and in 63 cases with no ticks (*N* = 103 capture events), resulting in 618 data points with presence/absence data, based on the estimated attachment days (103 × 6 days back in time) of a total of 56 ticks that had been feeding for ≤ 96 h, according to the C/S index. To explain host-seeking activity in female ticks, the retained model with the lowest number of parameters and lowest AIC_c_ included only the temperature 2 days prior to the estimated attachment day, and the second-best model also included precipitation as an interaction term at the day of attachment (Fig. 2, Tables 4, 5). For the nymphs, the null model had the lowest AIC_*c*_, and thus the probability of finding a nymph was not explained by any of the tested models (Table 4) (Additional file 1: Appendix 3).

**Table 5:** The most parsimonious logistic regression model explaining the findings of a female *Ixodes ricinus* on a roe deer, 2 days prior to the estimated attachment day (EAD, −2), from December to February 2013–2015, in southeast Sweden

Coefficients	Estimate	Std. error	z-value	*P*( >|t|)
Intercept	−1.952	0.170	−11.48	< 0.0001
Temperature (EAD, -2)	0.096	0.072	1.34	0.181

## Discussion

To the best of our knowledge, this is the first study to document active and host-seeking ticks during winter in Scandinavia. Attached nymphs, females and males were found on re-examined roe deer, indicating that these ticks were active between December and February. Both nymphs and females were attached and parasitizing, and their preferred sites were ears and groin, respectively. The most important weather factor regulating host-seeking activity for the females was air temperature.

We found ticks to be active at much lower temperatures than previously reported (≥ 5 °C, [21]), and clearly at temperatures well below 5 °C. Previous studies on the questing behaviour of *I. ricinus* females showed an attraction to +37 °C water tubes and sheep wool odour, and such attraction was also observed for the odour of dog, rabbit, cow and horse [39]. Consequently, roe deer odour and body temperature probably activate host-seeking behaviour in the same way. Further, roe deer bed sites during winter are found to be selected in relation to ambient temperature and wind, and thus local bed site temperatures are slightly higher than the surroundings [40]. In such bed sites, it seems plausible that ticks could also initiate an opportunistic activity to encounter a host during winter in Scandinavia, as shown in the present study. Therefore, we do not necessarily believe that ticks are actively questing in the sense that they climb onto the vegetation waiting for a host, but rather that they could be activated from quiescence by the warmth and odour radiating from a resting deer. Our weather variables are thus proxies for the actual microclimate in the proximity of deer bed sites. As a result, we believe that our result of increased tick activity at higher temperatures and precipitation is correct, but that our estimated thresholds for minimum activity temperature should be taken with caution. The additive effect of precipitation is puzzling. Desiccation could be one reason for this observation [17, 19], although we do not know whether humidity is a factor limiting ticks during winter. Even if our analysis of collinearity between precipitation and temperature was not significant (*r*_s_ = 0.019, *P* = 0.73), other weather conditions related to rain or snow that we did not investigate could be. It is thus more likely that the observed effect of precipitation in winter is related to higher temperatures since the air then can hold enough moisture for rain or snow. As temperatures drop significantly below freezing, the air becomes drier, reducing the likelihood of precipitation in general (SMHI).

An experimental study of questing behaviour in *I. ricinus* demonstrated that as many as 80% of Scottish ticks were questing at 10 °C, whereas the proportion of questing ticks originating from southern France was 40% at the same temperature [7]. Thus, it has been suggested that ticks in cooler climates are adapted to quest at lower temperatures than ticks in warmer climates because they have a shorter season to find a host [7]. In an evolutionary context, selection acts on an individual level, and if a behaviour is unfavourable and constantly failing, it will eventually disappear as a trait from the population. Still, if the alternative is to die of starvation or for other reasons, winter activity might be a sign of desperation. If most of the ticks we found are of local origin, and given that we have observed winter-active ticks in two populations for 3 years, we could likely infer that at least some winter-feeding ticks survive and eventually reproduce. A behaviour, when successful, results in a tremendous advantage in relation to their conspecifics that remain in quiescence throughout the whole winter with an uncertain outcome. Thus, finding a host even at low temperatures seems to be occasionally beneficial, simply because the behaviour obviously is maintained in the population. However, we have no information about the genetic adaptations or origin of our found winter-active ticks, and we acknowledge that our reasoning could be considered highly speculative. Still, our findings that ticks were active during winter in Sweden at even lower temperatures, and that activity varied with temperature, seem to corroborate those of Gilbert et al. [7].

Mejlon [19] previously suggested findings of ticks on animals in winter as an overwintering strategy. Even though we investigated individual animals repeatedly throughout the winter we cannot completely discard that possibility since we did not examine the whole animal. Because of time constraints while handling wild and un-sedated animals, we could only examine the head, ears, neck, groins and belly. The attached and feeding ticks that were found during handling could theoretically have remained for several months unattached in other parts of the animal that we did not examine. Still, according to Kiffner et al. [41, 42], approximately 50–70% of the ticks are found on the head, neck and belly, depending on life stage. Therefore, it is unlikely that the majority, if any, of the ticks discovered suddenly appeared and started to parasitize in the examined parts of the hosts after several weeks or months of overwintering on other unexamined body parts.

Determining the abiotic conditions activating tick host-seeking behaviour is crucial in the forecasting of climate change consequences and predicting future tick population dynamics [43]. From our results, we predict that at an air temperature of −5 °C, the probability of finding an attached tick on a roe deer is more than 8.0%, and that probability increases to almost 20% if the air temperature increases to 5 °C. These results are likely to apply to major parts of southern Scandinavia between December and March. During the warmest winter, 2014/2015 (mean 3.6 °C, vs 1.2 °C for both of the other two winters), we found the highest proportion of attached ticks per examined roe deer (48%, compared to 26% and 33% in 2013/2014 and 2015/2016, respectively), which clearly indicates that *I. ricinus* are more active during warmer winter conditions.

Belozerov et al. [44] described tick winter inactivity as a behavioural diapause and a temporary suppression of the host-seeking behaviour. We rather suggest the temporary periods of inactivity as temperature-regulated quiescence [18]. Our results thus corroborate the findings by Dautel et al. [45], where ticks in Berlin were found to be active during mild winters, supporting the contention that *I. ricinus* enters quiescence rather than a diapause. As an ectotherm, *I. ricinus* is particularly sensitive to surrounding environmental conditions [46]. However, *I. ricinus* can survive temperatures below freezing for short periods, and it is not lethal for active ticks if the temperature decreases below 0 °C for a short period of time [22], particularly since cold resistance and activity increase during infection with *A. phagocytophilum*, as has been demonstrated in *Ixodes scapularis * [26]. Still, the developmental cycle is much shorter in Southern Europe than in Central and Northern Europe, and one explanation is that ticks are active throughout the winter. Finally, it is well known that pathogens can manipulate the behaviour of their hosts and vectors [25]. Whether this phenomenon, at least to some degree, could explain the winter activity of *I. ricinus* in our study is unknown but is of major interest to investigate in future research.

The present study is the first attempt to investigate winter activity and feeding in the Northern Hemisphere. In conclusion, since we have documented active and blood-feeding ticks during several winters in two contrasting northern environments, we believe this behaviour to be a common phenomenon. Thus, if winter-active ticks become increasingly successful in the future it may have important consequences for the epidemiology of tick-borne pathogens.

## Supplementary Information

**Additional file 1: Appendix 1.** Data from three consecutive winters at Bogesund Research Area. First date in each week is given. “Low average” and “high average” represent the average for lowest and highest temperature from the indicated week. “Weeks high” represents the highest measured temperature for the indicated week. “Tot ticks” is the total number of ticks found at all captured roe deer that week. “Ticks/roe deer” represents the total number of ticks divided by the number of captured roe deer that week. **Appendix 2.** Data from the winter 2015/2016 at Grimsö Wildlife Research Area. First date of each week is given in the table. “Low average” and “high average” represent the average for lowest and highest temperature from the present week. “Weeks high” represents the highest measured temperature for the present week. “Tot ticks” is the total number of ticks found at all captured roe deer that week. “Ticks/roe deer” represents the total number of ticks divided by the number of captured roe deer that week.** Appendix 3.** Estimated snow depth and estimated attachment day of ticks detected on examined roe deer during December to February, 2013 - 2015 in South central Sweden

## Data Availability

The datasets used in this study are available from the corresponding author on reasonable request.
