# Supplementary material for: Winter activity of Ixodes ricinus in Sweden
Source: Parasit Vectors. 2023 Jul 10;16:229. doi: 10.1186/s13071-023-05843-9 (PMC10334512; doi:10.1186/s13071-023-05843-9)
Supplement: Supplementary file 1 — Additional file 1: Appendix 1. Data from three consecutive winters at Bogesund Research Area. First date in each week is given. “Low average” and “high average” represent the average for lowest and highest temperature from the indicated week. “Weeks high” represents the highest measured temperature for the indicated week. “Tot ticks” is the total number of ticks found at all captured roe deer that week. “Ticks/roe deer” represents the total number of ticks divided by the number of captured roe deer that week. Appendix 2. Data from the winter 2015/2016 at Grimsö Wildlife Research Area. First date of each week is given in the table. “Low average” and “high average” represent the average for lowest and highest temperature from the present week. “Weeks high” represents the highest measured temperature for the present week. “Tot ticks” is the total number of ticks found at all captured roe deer that week. “Ticks/roe deer” represents the total number of ticks divided by the number of captured roe deer that week. Appendix 3. Estimated snow depth and estimated attachment day of ticks detected on examined roe deer during December to February, 2013 - 2015 in South central Sweden [file 13071_2023_5843_MOESM1_ESM.pdf]

Appendix 1. Data from three consecutive winters at Bogesund Research Area. First date in each week is given. Low average and high average represent the average for lowest and highest temperature from the indicated week. 'Weeks high' represent the highest measured temperature for the indicated week. Tot ticks = the total number of ticks found at all captured roe deer that week; Ticks/roe deer = the total number of ticks divided by the number of captured roe deer that week.

Season	First date	Low average	High average	Weeks high	Tot ticks	Ticks/ roe deer	Nymphs/ roe deer	Females/ roe deer	Males /roe deer (not *in copula*)
13/14	2013-12-14	2.9	6.2	8.2	10	1.11	0	0.56	0.56 (1)
13/14	2013-12-21	3.6	6.5	7.5	2	0.5	0	0.5	0
13/14	2014-01-11	-7.1	-3.9	-1.9	2	0.2	0	0.2	0
13/14	2014-01-18	-5.2	-3.1	-2	0	0	0	0	0
13/14	2014-01-25	-3.6	-0.9	0.9	4	0.4	0.2	0.2	0
13/14	2014-02-01	0.5	2.8	4.7	0	0	0	0	0
14/15	2014-12-14	0	3.6	5.4	8	0.53	0.13	0.4	0
14/15	2015-01-04	-2	2.1	4.8	0	0	0	0	0
14/15	2015-01-11	0.4	4.2	6.1	1	0.08	0.08	0	0
14/15	2015-01-18	-2.5	0.7	2.3	2	1	1	0	0
14/15	2015-01-25	-0.7	2	3.6	5	0.56	0.56	0	0
14/15	2015-02-08	0	4.1	6.8	4	1	0.25	0.75	0
14/15	2015-02-15	1.1	4.3	8.4	2	1	1	0	0
14/15	2015-02-22	0.8	4.6	7.6	33	1.27	0.12	0.62	0.54 (3)

15/16	2015-12-14	2.8	7	12.7	32	2.91	0.27	1.73	0.91
15/16	2015-12-21	1.9	6.2	11.6	38	2.53	0.53	1.4	0.6
15/16	2015-12-28	-2.8	0.4	2.8	39	4.33	0.22	2.78	1.33
15/16	2016-01-04	-9	-4.2	3	13	0.87	0.4	0.4	0.07
15/16	2016-01-11	-11.2	-6.5	2	2	0.17	0	0.08	0.08
15/16	2016-01-18	-7.3	-2.7	1.7	1	0.06	0.06	0	0
15/16	2016-02-01	-0.2	4.6	7.8	3	0.15	0.1	0.05	0
15/16	2016-02-08	-1.1	2.2	5.5	4	0.19	0	0.14	0.05
15/16	2016-02-15	-1.6	2.2	3.6	17	0.74	0	0.57	0.17
15/16	2016-02-22	-3	2.9	4.2	4	0.11	0	0.11	0

Appendix 2. Data from the winter 2015/2016 at Grimsö Wildlife Research Area. First date of each week are given in the table. “Low average” and “high average” represent the average for lowest and highest temperature from the present week. “Weeks high” represent the highest measured temperature for the present week. “Tot ticks” is the total number of ticks found at all captured roe deer that week and “Ticks/roe deer” represent the total number of ticks divided with the number of captured roe deer that week.

Season	First date	Low ave-rage	High ave-rage	Weeks high	Tot ticks	Ticks/ roe deer	Nymphs/ roe deer	Females/ roe deer	Males /roe deer (not *in copula*)
15/16	2015-12-14	-2.7	2.3	9.8	0	0	0	0	0
15/16	2016-01-04	-16.4	-9.7	-2.7	1	0.13	0	0.13	0
15/16	2016-01-11	-13.9	-8.1	-1.8	0	0	0	0	0
15/16	2016-01-18	-13.5	-6.1	-0.8	1	0.17	0	0.17	0
15/16	2016-02-01	-2.4	2.6	5.3	0	0	0	0	0
15/16	2016-02-08	-2.8	0	4.5	0	0	0	0	0
15/16	2016-02-15	-7.1	-0.7	1	0	0	0	0	0
15/16	2016-02-22	-7.5	2.2	3.9	1	0.33	0	0.33	0

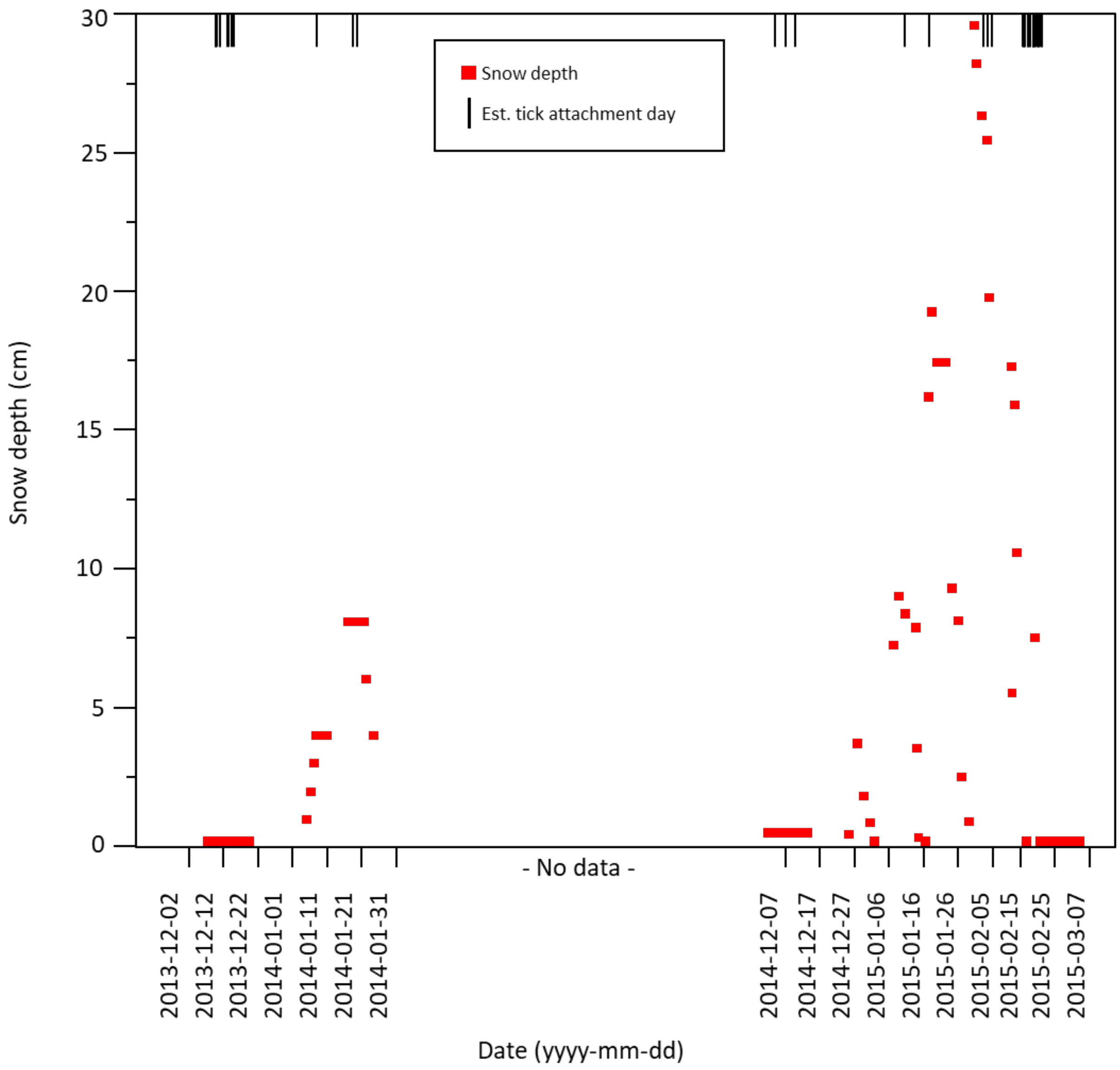

Appendix 3. Estimated snow depth and estimated attachment day of ticks detected on examined roe deer during December to February, 2013 - 2015 in South central Sweden
